# Report of the Commissioner of Education for the Year 1894-95

**Published:** 1897-08

**Authors:** 


### Report of the Commissioner of Education for the Year 1894-95.

We learn from the report that there were in the United States, 1894-95, 145 Schools of Theology, with 906 teachers, 8,050 students, and 1,598 graduates. There were 72 Law Schools, 8,950 law students, 2,717 graduates, and 604 teachers. There were 151 "Medical Schools," of whicn 113 were regular, 20 homœopathic, 9 electic, 2 physio-medical, and 7 graduate. Medical students numberd 22,887, of whom 18,660 were in regular schools, 1,875 in homœopathic, and 732 in electic; the graduates numbered 4,827, and teachers 3,907. There were 45 Schools of Dentistry, with 5,437 students, 1,297 graduates, and 968 teachers. 39 Schools of Pharmacy, with 3,858 students, 1,067 graduates, anb 317 teachers. There were 9 Veterinay Schools, 132 teachers, 474 students, and 155 graduates. The Schools for the training of Nurses numbered 131, 3,985 students, 1,498 graduates. Of the 22,887 medical students, 1,413 were women; of the 8,950 law students, 65 were woman. "Although about one-third of the Theological Schools made no report as to the value of grounds and buildings, and the amount of endowment funds, the remaining schools reported grounds and buildings to the value of $12,057,747, and endowment funds to the amount of $16,083,683, while benefactions for the one year aggregated over $1,000,000. The endowment funds reported from medical schools aggregated only $749,992, and although these figures are below the real amount it nevertheless remains true that medical schools are practically without endowments. From law schools the amounts reported were so small that they were not tabulated. Although medical schools have such meagre endowments they own much valuable property, frequently near the center of large cities. From the schools reported the value of property is $8,-130,422.

